# Effect of cognitive bias modification training on body image dissatisfaction in adolescents with anorexia nervosa or depression—a pilot feasibility randomized controlled crossover study

**DOI:** 10.3389/fpsyg.2025.1655064

**Published:** 2025-09-26

**Authors:** Eva Glombitza, Piers L. Cornelissen, Martin J. Tovée, Tanja Legenbauer

**Affiliations:** ^1^Faculty of Medicine, Ruhr University Bochum, Bochum, Germany; ^2^LWL University Hospital for Child and Adolescent Psychiatry, Medical Faculty, Ruhr University Bochum, Bochum, Germany; ^3^Department of Psychology, Faculty of Health and Life Sciences, Northumbria University, Newcastle upon Tyne, United Kingdom

**Keywords:** anorexia nervosa, depression, adolescents, cognitive bias modification, body image dissatisfaction

## Abstract

**Introduction:**

Body image disturbance presents transdiagnostically with an impact on the development and maintenance of psychiatric disorders. Addressing body image disturbance, a cognitive bias modification training (CBM) was developed using a two-alternative forced choice task (2-AFC) to alter patients’ individual perceptual boundary between what they classify as a fat versus a thin body. This pilot study aimed to evaluate, for the first time, the paradigm’s feasibility and efficacy in adolescents in a clinical context.

**Methods:**

This pilot study included adolescent inpatients aged 13–17 years diagnosed with (atypical) anorexia nervosa (*n* = 12) or depression (*n* = 17), representing two prevalent disorders in adolescence. The 2-AFC task was tested in this population for the first time. Designed as a randomized crossover trial, patients underwent a 4-day intervention with corrective feedback and a 4-day control with confirmatory feedback. Psychometric measures assessing body image disturbance, depressive symptoms, and general psychopathology were collected at the beginning of the training and 10 days afterward (day 1, 15, 29).

**Results:**

Mixed ANOVAs showed that the 2-AFC CBM paradigm significantly shifted the categorical boundary over 10 days, altering patients’ individual perceptual boundary but did not improve the body image specific psychometric measures. Linear regression indicated training effects on diagnosis-specific characteristics, and *t*-test comparisons revealed improved depression-specific symptoms for the depression group. The control condition had a non-neutral effect and shifted the individual boundary to a lower body mass index (BMI), particularly in patients with anorexia.

**Discussion:**

These findings confirm the feasibility and effectiveness of the 2-AFC CBM paradigm in adolescent inpatients transdiagnostically with further larger randomized controlled effectiveness trials required. The study suggests including normal-weight patients with anorexia nervosa only and not using confirmatory feedback as a control condition, but as an orientation and confirmation of a healthy weight limit.

## 1 Introduction

Adolescence is a vulnerable period for body image development due to cultural, physical, psychological, and social changes (Voelker et al., 2015). Studies indicate that most girls aged 11–15 engage in weight control strategies, with over 40% perceiving themselves as overweight (Studienverbund Deutschland et al., 2020). Perceptual biases are evident, as children and adolescents often underestimate the body size of young women by over two BMI units (e.g., Schuck et al., 2018).

Body image is shaped by multiple contextual influences, including digital transformation (Saiphoo and Vahedi, 2019; Holland and Tiggemann, 2016; Mazzeo et al., 2024), family (Kluck, 2010), and the social environment (Forney et al., 2019). Such factors can contribute to the development of body image dissatisfaction. For instance, exposure to and comparison with beauty ideals portrayed in social media are associated with thin-ideal internalization, body surveillance, an increased drive for thinness, and body dissatisfaction (Mazzeo et al., 2024; Cohen et al., 2017; Sanzari et al., 2023). In addition, online health information, which often contains misinformation, may negatively influence adolescents’ health-related attitudes (Swire-Thompson and Lazer, 2020), whereas mobile health applications and digital interventions can both positively and negatively affect body image outcomes (Mahon and Seekis, 2022) and health-promoting behaviors conceptually related to proactive health behavior (Babbott et al., 2023; Beintner et al., 2019; Stice et al., 2007).

Body image disturbances are recognized as transdiagnostic factors in depression, predicting depressive symptoms (Sharpe et al., 2018; Stice et al., 2000). Risk factors for the development of depression include body dissatisfaction, negative body evaluations, overvaluation of weight and shape (Sharpe et al., 2018), body-focused attention (Murray et al., 2018) and weight-loss strategies (Rawana, 2013). Integrating body image interventions in depression treatment has therefore been recommended (Rawana, 2013). No studies have been identified that address body image disturbances in depression treatment, though such interventions have reduced depressive symptoms in eating disorder patients (Delinsky and Wilson, 2006).

Cognitive-behavioral therapies for adolescents show modest success, with only half of patients achieving lasting remission (Crone et al., 2023; Dalle Grave et al., 2021; Vogel et al., 2021). Thus, effective age-appropriate treatment strategies are needed. Previous studies have shown that digital interventions in adolescents and young adults can effectively improve specific body image outcomes (Mahon and Seekis, 2022) and that body judgments are represented as categorical perceptions (e.g., healthy vs. unhealthy, thin vs. fat, Tovée et al., 2012). The position and malleability of this boundary is influenced by body image concerns, with higher concerns lowering the thin-fat BMI boundary towards lower BMI levels (Gledhill et al., 2017; Cornelissen et al., 2022). A two-alternative forced choice (2-AFC) cognitive bias modification (CBM) paradigm was developed to address this downward shift, and has been shown to shift body categorizations, reduce eating concerns, and improve body image (Gledhill et al., 2017; Irvine et al., 2020; Szostak, 2018). Targeting perceptual processes underlying body dissatisfaction, this paradigm may be described as proactive, though not in the sense of health behavior. However, studies testing this paradigm and examining these effects in clinical adolescent samples are still lacking.

This study examines the feasibility and efficacy of the 2-AFC CBM paradigm in adolescent inpatients as an adjunct to conventional inpatient treatment. Focusing transdiagnostically on two most relevant disorders of adolescents, patients diagnosed with (atypical) anorexia nervosa (AN) or depression (DEP) were included. Using a crossover design, all patients received both intervention and control conditions, allowing for disorder-specific and comparative analyses. It is hypothesized that the 2-AFC task with corrective feedback will improve body image disturbances and general psychopathology in adolescent female patients with AN or DEP compared to confirmatory feedback. Additionally, the study explores whether patients with AN benefit more from the training than those with DEP.

## 2 Method

### 2.1 Study design

For this counterbalanced, randomized, cross-over designed study, the CONSORT guidelines (Eldridge et al., 2016) were followed to examine the pilot feasibility, acceptability and efficacy of the 2-AFC CBM transdiagnostically for adolescents. The study protocol was approved by the ethics committee of the medical faculty of the Ruhr-University in Bochum (Reference number: 207134). Informed consent was obtained from all patients and caregivers.

### 2.2 Patients

Patients were recruited from the inpatient setting of a university hospital for child and adolescent psychiatry in Germany. The inclusion criteria required patients to be female, aged 13–17 years and diagnosed with depression (*n* = 17) or (atypical) anorexia nervosa (*n* = 12) according to the International Classification of Diseases, 10th Revision (ICD-10, Weltgesundheitsorganisation, 2022) as is standard in the German healthcare system. The ICD-10-based clinical diagnoses were applied by experienced clinicians. Diagnostic procedures were routinely conducted whenever symptom-related abnormalities were observed, and comorbid conditions were systematically assessed to examine potential exclusion criteria. Depression was selected as one diagnostic group because it is a frequent disorder in adolescence, studies highlight the role of body image dissatisfaction, and therapeutic options addressing this aspect remain limited. Other diagnostic groups with transdiagnostic body image problems were either not available in sufficient numbers at the study site or were too acute for participation. (Atypical) Anorexia nervosa was chosen for the eating disorder group because body image symptomatology is particularly pronounced in this disorder and as the most frequent eating disorder requiring inpatient treatment, it represented the largest eating disorder cohort in the clinic. Atypical anorexia nervosa is defined as an eating disorder (ED) that meets almost all criteria for anorexia nervosa. In the present sample, *n* = 7 patients were diagnosed with anorexia nervosa and *n* = 5 with atypical anorexia nervosa; three of the ED patients also fulfilled criteria for comorbid depression. In these cases, anorexia nervosa was considered the primary diagnosis and served as the distinguishing criterion separating the ED group from the DEP group. All screened and included patients in the DEP group had moderate depression, the most diagnosed level in Germany (Stahmeyer et al., 2022). Patients with acute suicidality, severe depressive symptoms, BMI > 25 kg/m^2^, autism spectrum disorder (ASD), IQ < 70, schizophrenia or other psychotic disorders, uncorrected vision impairments or insufficient German language skills of the patient or caregivers were excluded. These criteria ensured focus on the relevant diagnoses and avoided ethical concerns regarding experimental participation.

**Figure 1:**
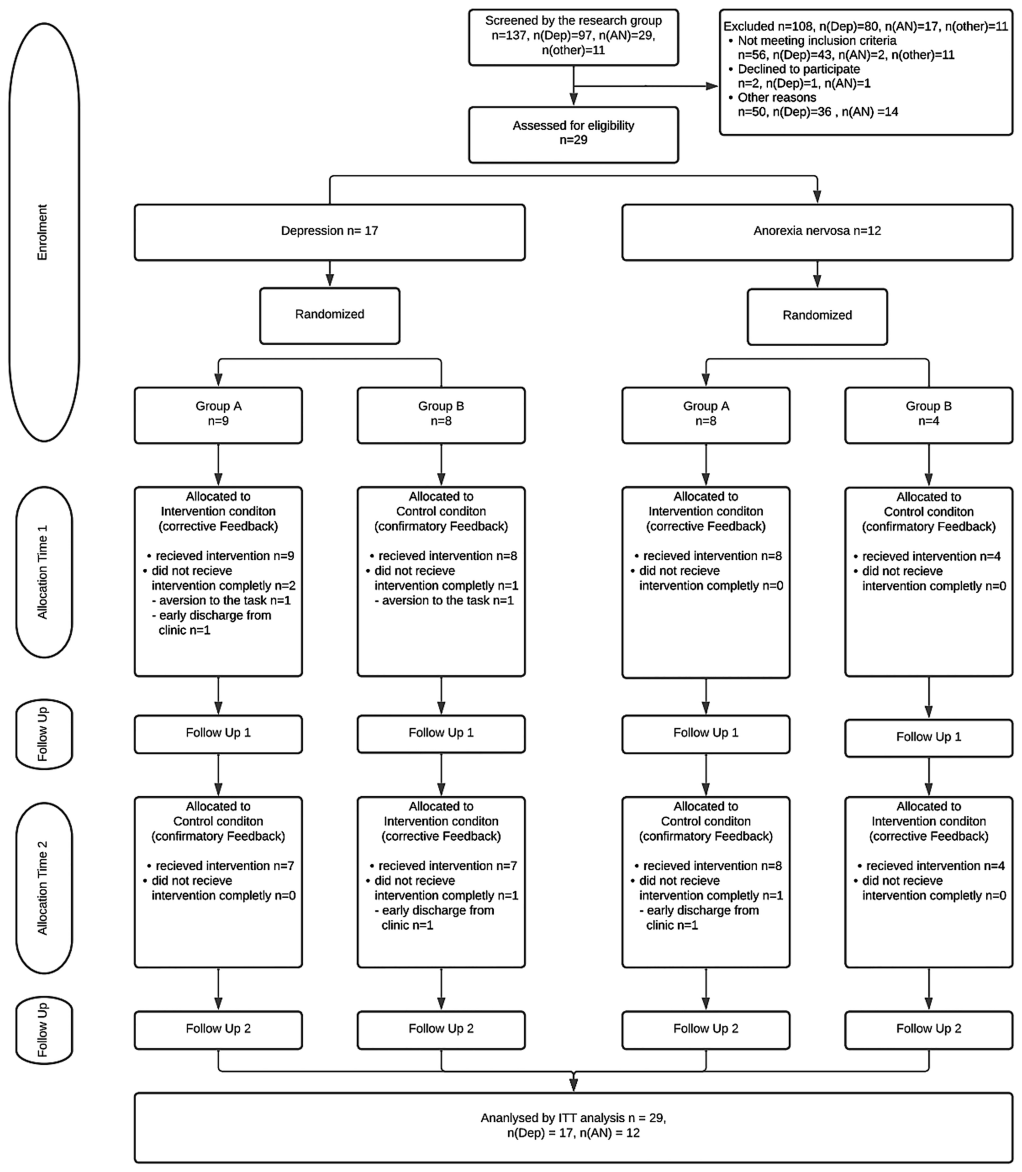
Consort flow chart. Dep = Depression Group, AN = Anorexia group, ITT = Intention-to-treat analysis.

Power analysis (G*Power; Faul et al., 2009) based on data from previous studies (Gledhill et al., 2017) indicated a required sample size of *n* = 24 (*d* = 1.04, *α* = 5%, power = 80%). To account for potential dropouts, the target sample size was set at *n* = 30. Figure 1 shows the corresponding CONSORT flow chart.

### 2.3 Intervention

This study used a 2-AFC/CBM paradigm by Gledhill et al. (2017). Pre-study interviews showed adolescents compared themselves to young adult bodies, so computer-generated images of a young adult in underwear were used. Fifteen photorealistic avatars with BMIs from 15 to 33.7 were shown, each presented three times to establish patients’ individual baseline by categorizing each avatar as “thin” or “fat” using their keyboard. Patients were randomized into two groups (A/B) in a counterbalanced design, receiving intervention and control conditions in different orders. Group A received the intervention first and then the control condition, while Group B received the control condition first and then the intervention. Training occurred over two four-day blocks (Days 1–4 and 15–18), with daily pre- and post-baseline assessments and six blocks of 31 trials. Avatars with extreme BMIs appeared once per block, while moderate BMIs appeared more frequently, based on prior research (Gledhill et al., 2017). In addition to baseline measures before and after each training session, questionnaires were administered at the beginning of the training and 10 days afterward (day 1, 15, 29). Day 15 thus served both as the 10-day follow-up after the first block and as the baseline of the second training block. In the intervention condition, feedback aimed to shift the categorical boundary (Catbound) two BMI points higher than baseline, correcting “old” boundaries with messages like “Wrong! This body was thin” or “Wrong! This body was fat.” In the control condition, participants completed the same assessments as in the intervention condition, but the confirmatory feedback was aligned with each patient’s baseline categorial boundary.

### 2.4 Randomization

Patients were randomized 1:1 using the chit method (Singh, 2006) by study staff, with results securely stored for allocation concealment. Patients were blinded to their intervention group due to baseline-based feedback, but study staff were not blinded.

### 2.5 Measures

#### 2.5.1 Primary outcome

Body dissatisfaction, attitude, and figure perception were measured using the German version of the Body Shape Questionnaire (BSQ) (Cooper et al., 1987), a 34-item self-report tool on a 6-point Likert scale (1 = never, 6 = always), with higher scores (34–204) indicating greater body dissatisfaction. The German version of the BSQ has demonstrated excellent internal consistency (Cronbach’s *α* > 0.94) and strong construct validity. Its sensitivity to change makes it suitable for research and therapy diagnostics in women (Pook et al., 2002).

#### 2.5.2 Secondary outcomes

ED- and DEP-specific measures were assessed using German versions of the respective questionnaires. A 2-AFC validation check was performed using pre- and post-training categorical boundary assessments. The following secondary outcomes were pre-registered as relevant to the hypotheses, with additional questionnaires analyses available in the supplementary materials.

PHQ-9 (Patient Health Questionnaire, Spitzer et al., 1999; Löwe et al., 2002): Assesses depressive symptoms over 2 weeks with 9 items rated from 0 (not at all) to 3 (nearly every day) with good sensitivity and specificity compared to the Structured Clinical Interview for DSM-IV (Gräfe et al., 2004) and suitable for measuring DEP severity (Kroenke et al., 2001). The questionnaire has demonstrated good internal consistency (Cronbach’s *α* = 0.89; Indu et al., 2018) and solid construct validity (Martin et al., 2006).

ANIS (Anorexia Nervosa Inventory for Self-Assessment): Measures deviant eating behaviors and attitudes via 31 items across six subscales on a 6-point scale (0 = not at all, 5 = very much). The ANIS has been validated for screening, diagnosis, and treatment monitoring in women, with confirmed convergent validity with the EDI (Fichter and Keeser, 1980; Rathner and Rumpold, 1994) and high internal consistency (Cronbach’s α = 0.70–0.88; Rathner and Rainer, 1998).

2-AFC Pre- and Post-Training Baseline: Body perception bias was evaluated with 15 computer-generated avatars (BMI: 15–33.7) shown three times each before and after training. The individual baseline at which BMI a stranger’s body was categorized as “thin” or “fat” was estimated following a modified method by Penton-Voak et al. (2013).

### 2.6 Data analysis

Statistical analyses were performed using SPSS 28. The sample was characterized using descriptive statistics and following the Intention-to-treat protocol, missing data were imputed by means of linear regression models. For each variable with missing data, a regression model was specified in which significant correlations between the available questionnaire scores, missing questionnaire scores, and Catbound values served as predictors to preserve the multivariate structure of the data.

To check the assumption of negligible carryover effects pre-tests were conducted using Welsh t-tests and Mann–Whitney U-tests comparing within-subject sums of the results from both periods (Wellek and Blettner, 2012). Treatment effects were tested with Welch t-tests or Mann–Whitney U-tests based on parametric conditions, comparing within-subject outcome differences between periods. A mixed ANOVA analyzed the effect of the 2-AFC task with corrective feedback on the BSQ, using baseline as covariate, time as within-subject factor, and diagnosis and cross-over group as between-subject factors. The direct effect on general psychopathology was examined with multiple mixed ANOVAs using the same corresponding covariate and factors. Differences between each training day’s post-baseline and the pre-baseline were plotted against zero by diagnosis and cross-over group. Linear regressions examined the relationship between post-training Catbound values and follow-up questionnaire scores by diagnosis. Treatment effects, including 95% confidence intervals, *p*-values, and Cohen’s d effect sizes, were calculated separately for each diagnosis using Welch *t*-tests or Mann–Whitney *U*-tests. Non-parametric effect sizes were converted following Dunlap (1994).

## 3 Results

### 3.1 Descriptive statistics

Table 1 summarizes the sample characteristics of the DEP and AN group before the trial and the Welsh *t*-test referring to pre-measurement comparisons.

**Table 1:** Baseline characteristics of patients with depression and anorexia nervosa.

Variable	Depression		Anorexia nervosa		DEP vs. AN
	*M*	*SD*	*M*	*SD*	*p*
Age (years)	16.24	1.11	15.07	1.45	0.029
BMI	21.07	3.30	18.12	1.29	0.003
ANIS	2.28	1.09	3.16	0.30	0.005
BSQ	72.73	42.67	117.50	21.18	0.001
PHQ	16.72	6.39	17.75	4.54	ns

BMI = body mass index adjusted for age and gender, normative range between 18.5–25, ANIS = Anorexia nervosa Inventory, BSQ = Body Shape Questionnaire, PHQ = Patient Health Questionnaire, M = mean, SD=Standard deviation, DEP vs. AN p = two- tailed *p-*value for unpaired *t*-test comparing means of the DEP with means of the anorexia nervosa group.

The groups differed in relation to age and BMI, with the AN group presenting younger and with lower BMI’s. As consistent with their diagnoses, patients with AN showed significantly higher scores than patients with DEP on BSQ and ANIS. Furthermore, the level of DEP measured with the PHQ was comparable between the groups. The later indicating severe symptoms of DEP in both groups. Baseline comparisons between the randomization groups revealed no significant differences. When separating by diagnosis, no differences emerged within the depression group, whereas in the eating disorder group a small baseline difference was found for the BSQ (*p* = 0.049).

### 3.2 Validation check

#### 3.2.1 Pre-test for carryover effects

Pre-tests checking the assumption of negligible carryover effects and training effects were conducted revealing no evidence of relevant carry-over effect. Details on these analyses are included in the additional files (Appendix Table A1).

#### 3.2.2 Training effect

**Figure 2:**
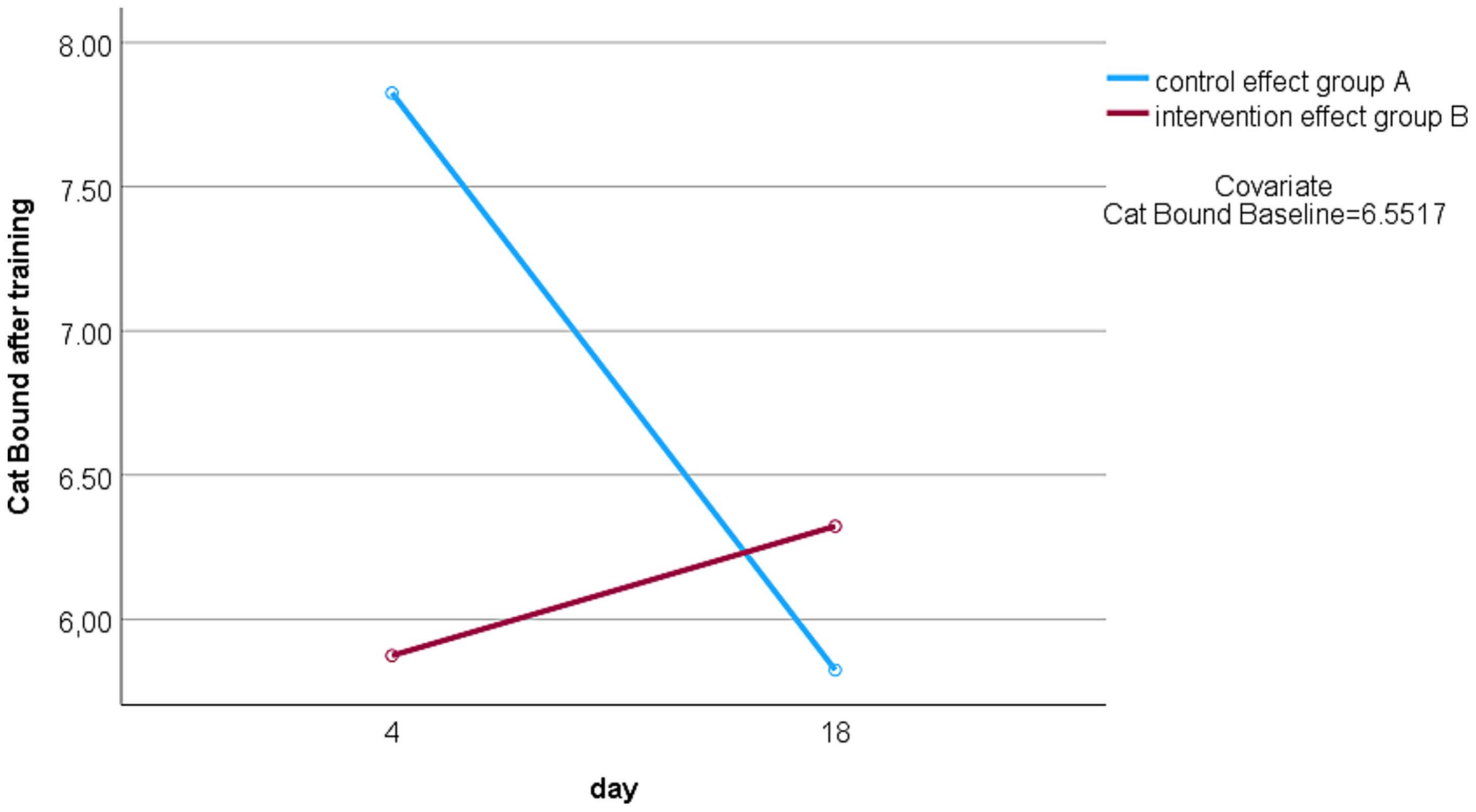
Change Catbound over time. Catbound after training on day 4: group A = end of intervention condition, group B = end of control condition; Catbound after training on day 18: group A = end of control condition, group B = end of intervention condition.

Comparing the two study periods, treatment effects could be found for the Catbound in the 2-AFC task, changing significantly directly after the training *t(25,561) = 4,011; p < 0.001* and at follow up *U = 32; Z = –3.145; p = 0.002.* A significant within-subject time*crossover interaction was found for the training’s effect on the Catbound (2-AFC baseline) when using post-training values (days 4 and 18) as dependent variables, *F(1,24) = 12.528, p = 0.002, ηp^2^ = 0.343*, and when including follow-up values after the washout period (days 15 and 29), *F(1,24) = 9.535, p = 0.005, ηp^2^ = 0.284*. Figure 2 shows exemplary the change of the Catbound after training on day 4 and 18 divided by cross over groups.

### 3.3 Hypothesis 1: Effect of the 2-AFC task on body image disturbance

No statistically significant effects of the 2-AFC task on body image disturbance were detected, with the BSQ showing non-significant interactions between time and baseline *F(1,24) = 0.523; p = 0.477*, time and crossover *F(1,24) = 0.3; p = 0.865* and time and diagnosis *F(1,24) = 0.134; p = 0.717.* The psychometric task performance means are presented in Table 2.

**Table 2:** Psychometric task performance in depression and anorexia nervosa groups.

Measure	Depression				Anorexia nervosa			
	Group A		Group B		Group A		Group B	
	*M*	*SD*	*M*	*SD*	*M*	*SD*	*M*	*SD*
ANIS 1	2.20	1.19	2.37	1.04	3.13	0.35	3.24	0.19
ANIS 2	2.11	1.24	2.43	0.93	2.94	0.60	3.48	0.37
ANIS 3	2.02	1.20	2.32	1.14	2.92	0.55	3.31	0.74
BSQ 1	69.33	46.56	76.54	40.66	110.25	21.54	132.00	11.92
BSQ 2	66.68	48.00	79.83	38.19	112.38	26.43	141.25	9.46
BSQ 3	68.34	48.62	74.95	40.02	108.90	23.27	141.00	14.00
PHQ 1	15.11	5.78	18.53	6.95	17.63	5.01	18.00	4.08
PHQ 2	14.37	6.67	16.90	6.69	17.38	4.03	19.25	2.87
PHQ 3	15.23	6.80	15.30	6.72	16.49	4.77	18.00	4.62
Catbound Pre	7.11	2.71	6.75	3.37	5.88	1.89	6.25	0.96
Catbound s 1	8.95	3.68	7.49	4.68	6.50	3.25	2.75	2.22
Catbound s 2	7.15	4.21	8.04	4.73	4.27	3.46	3.00	3.37
Catbound l 1	7.74	3.26	6.86	4.07	5.75	2.87	2.75	2.22
Catbound l 2	6.87	3.92	7.64	4.37	4.45	3.02	3.00	2.71

ANIS = Anorexia Nervosa Inventory for Self-Assessment; BSQ = Body Shape Questionnaire; PHQ = Patient Health Questionnaire; Catbound s = Catbound short; Catbound l = Catbound long; M = mean; SD = standard deviation.

### 3.4 Hypothesis 2: Effect of the training on eating and general psychopathology

Table 3 shows the direct effect of post-training Catbound values on the respective follow-up questionnaire scores, highlighting a significant training impact on ANIS and BSQ at Follow-Up 1 in both groups and at Follow-Up 2 in the DEP group.

**Table 3:** Regression analyses predicting outcomes by diagnosis.

Measure	Depression				Anorexia nervosa			
	*F*(1,16)	*p*	*R* ^2^	Adj. *R*^2^	*F*(1,11)	*p*	*R* ^2^	Adj. *R*^2^
ANIS 1	9.822	0.007	0.396	0.355	6.273	0.031	0.385	0.324
ANIS 2	10.124	0.006	0.403	0.363	4.878	0.052	0.328	0.261
PHQ 1	1.066	0.318	0.066	0.004	1.156	0.308	0.104	0.014
PHQ 2	0.944	0.347	0.059	−0.003	3.689	0.084	0.269	0.196
BSQ 1	8.711	0.010	0.367	0.325	11.427	0.007	0.533	0.487
BSQ 2	7.953	0.013	0.346	0.303	2.391	0.153	0.193	0.112

ANIS = Anorexia Nervosa Inventory for Self-Assessment; BSQ = Body Shape Questionnaire; PHQ = Patient Health Questionnaire.

### 3.5 Exploratory analyses: Comparison of treatment effects between diagnoses

**Figure 3:**
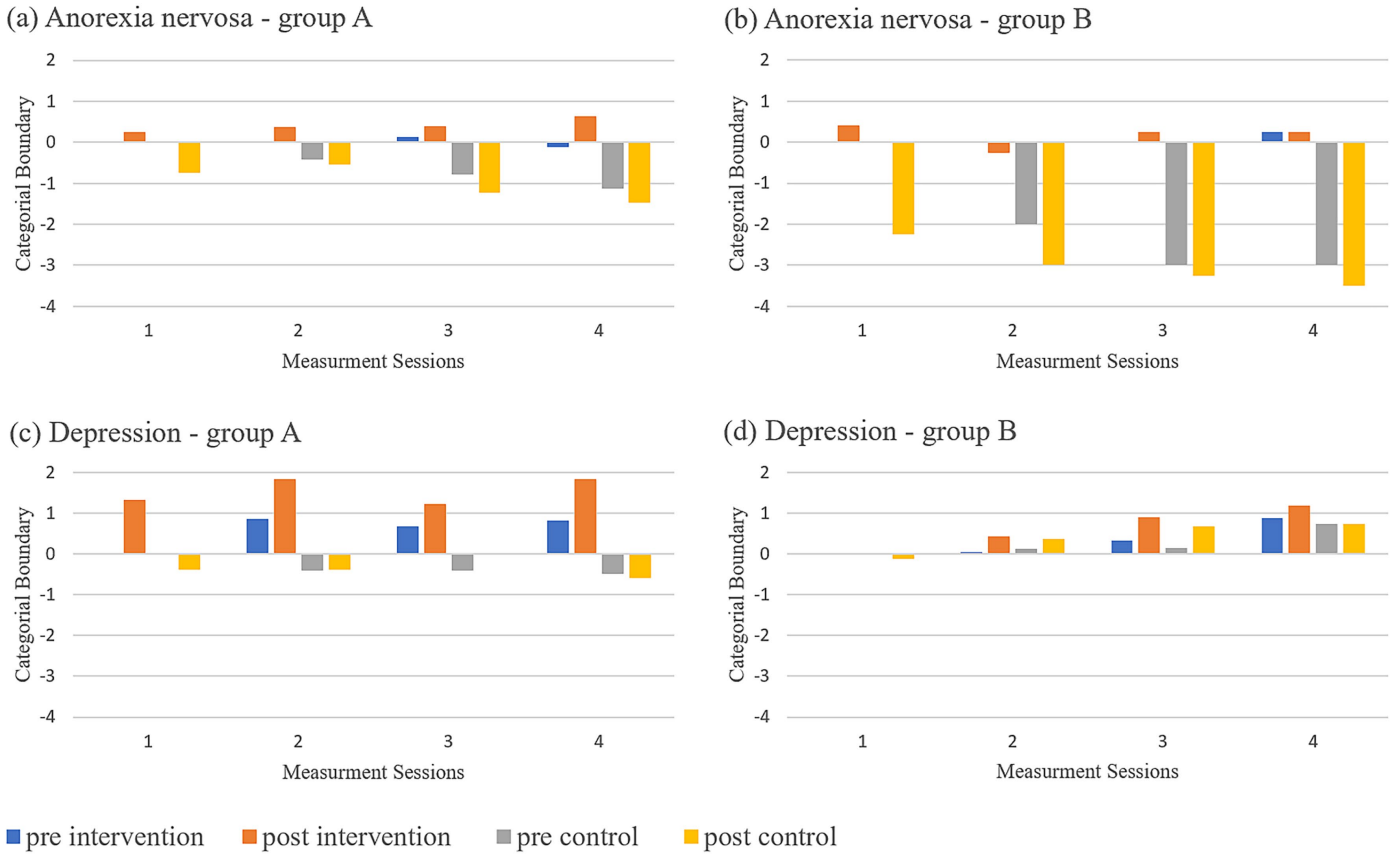
CatBound plotted against zero. Difference between CatBound after each training day and before the training sequence were plotted against zero separately for diagnosis and cross-over group for each day of training (1–4); pre intervention = before training with corrective feedback; post intervention = after training with corrective feedback; pre control = before training with confirmatory feedback; post control = after training with confirmatory feedback. **(a)** Anorexia nervosa - Group A, **(b)** Anorexia nervosa - Group B, **(c)** Depression - Group A, **(d)** Depression Group - B.

Change of Catbound: In addition to the significant within-subject interaction of time*crossover described above, a significant between-subject effect of diagnosis was found during training, *F (1,24) = 6.957, p = 0.014, ηp^2^ = 0.225*, and at follow-up, *F(1,24) = 6.596, p = 0.017, ηp^2^ = 0.216*. Figure 3 displays the difference between post- and pre-baseline for each training day, plotted against zero and separated by diagnosis and crossover group, with mean values in Appendix Table A2. The above analyzed training effect was observed in both diagnosis groups, pronounced in Group A (intervention first), where post-baseline increased. In Group A (DEP and AN) and particularly in Group B (AN), post-baseline decreased during the control condition, with this decrease accumulating over the 4 days of training. Patients with AN in group B showed the lowest post-baseline increase during the following intervention. The confirmed personal boundary (control condition) corresponded to a BMI of 24.65 kg/m^2^ (Group A, DEP), 23.42 kg/m^2^ (Group B, DEP), 22.17 kg/m^2^ (Group A, AN), and 22.79 kg/m^2^ (Group B, AN). Thus, patients with DEP classified most normal-weight avatars as thin, while patients with AN corrected avatars ≥22 kg/m^2^ as “fat.”

Welch t-tests/Mann–Whitney U-tests showed significant treatment effects for both diagnoses with high effect sizes during training (Post 4/8: DEP *t(14.96) = 2.706, p = 0.016, d = 1.31*; AN *t(8.05) = 2.852, p = 0.021, d = 1.57*) and at follow-up (Pre 5/9: DEP *U = 14.5, Z = –2.092, p = 0.036, d = 1.19*; AN *U = 3.5, Z = –2.185, p = 0.028, d = 1.62*).

Change of disorder specific psychopathology: A treatment effect was found in the DEP group for the PHQ, *t(13.58) = −2.434, p = 0.029, d = −1.198*. No significant changes were observed in the AN group. Table 4 presents the treatment effect analysis results for questionnaire and Catbound changes by diagnosis.

**Table 4:** Treatment effects in depression and anorexia nervosa groups.

Measure	Depression				Anorexia nervosa			
	Group A	Group B			Group A	Group B		
	*M*	*M*	*p*	Cohen’s *d*	*M*	*M*	*p*	Cohen’s *d*
ANIS	0.09	0.10	0.67*	0.24	0.03	0.18	0.72	−0.26
BSQ	−1.66	4.88	0.54*	0.32	3.48	0.25	0.72	0.22
PHQ	−0.86	1.60	0.03	−1.20	0.89	1.25	0.88	−0.11
Catbound s	1.80	−0.55	0.02	1.31	2.23	−0.25	0.02	1.57
Catbound l	0.87	−0.78	0.04*	1.19	1.30	−0.25	0.03*	1.62

ANIS = Anorexia Nervosa Inventory for Self-Assessment; BSQ = Body Shape Questionnaire; PHQ = Patient Health Questionnaire; Catbound s = Catbound short; Catbound l = Catbound long; M = mean; * = Mann–Whitney U-test (exact significance).

## 4 Discussion

The present randomized controlled pilot study examined the feasibility and efficacy of a 2-AFC CBM paradigm in adolescent AN and DEP inpatients as an adjunct to conventional treatment. In the crossover design, all patients received 4 sessions of corrective and 4 sessions of confirmatory feedback, with a 10-day washout period between the two conditions. Psychometric data were collected at baseline and 10 days post-training using various questionnaires.

The intervention significantly shifted the categorical boundary for perceiving a body as “fat” over 10 days, while the control condition decreased the Catbound, particularly in patients with AN. Post-training Catbound directly influenced AN and DEP-related characteristics. However, no significant effect on body image disturbance as indexed through the psychometric measures was found when all measurement points were combined. Both diagnostic groups benefited, with patients with DEP showing significant PHQ treatment effects.

### 4.1 Feasibility of the 2-AFC task

The 2-AFC task significantly shifted the categorical boundary for both diagnostic groups, with a stronger effect during training than at follow-up, indicating a decrease over time. Preliminary studies in adults showed that body perception can change through feedback and repetition (Gledhill et al., 2017; Irvine et al., 2020). This study replicated these findings in adolescents, demonstrating the task’s feasibility in this age group. However, the control condition had a non-neutral effect, not observed in adult studies (e.g., Gledhill et al., 2017). Adolescents are considered more suggestible than adults (Zimmermann et al., 2022) and emotionally vulnerable during puberty due to hormonal and brain changes (Konrad et al., 2013; Herpertz-Dahlmann et al., 2013), which may explain this.

Particularly in the AN group, the control condition with confirmatory feedback negatively shifted the boundary toward a lower BMI, accumulating over time and weakening the subsequent intervention effect in Group B. In the DEP group, the control condition had a positive effect with no weakened intervention effect. Patients with DEP had a higher boundary, labeling most normal-weight avatars as thin, while patients with AN labeled avatars ≥22 kg/m^2^ as “fat,” confirming and reinforcing their negative body image during the control condition. This may explain the different feedback effects between diagnoses.

The influence of “fat talk,” often underestimated, contributes to body dissatisfaction (Curtis and Loomans, 2014). The results emphasize the relevance of critical feedback. Feedback should serve as orientation and confirm a healthy normal weight limit, especially for suggestible patients who suffer from body image distortion.

### 4.2 Effect on disorder-specific characteristics

Previous studies indicated that modification of the categorical boundary is associated with body dissatisfaction (Cornelissen et al., 2022) and can be shifted through training programs (Gledhill et al., 2017; Irvine et al., 2020). In the present study, hypothesis-specific analyses examined this effect and showed multiple direct training effects on disorder-specific characteristics. In the AN group, this effect was limited to the first follow-up, likely due to the increased influence of the control condition for this diagnosis. A mixed ANOVA of all measurement points found no significant improvement in body image disturbance. This may be due to the control condition’s influence preventing significant improvement. Studies with adults showed reduced body shape and weight concerns (Gledhill et al., 2017; Irvine et al., 2020), including preclinical samples or patients with AN without a control group after leaving the intention-to-treat protocol (Gledhill et al., 2017). Thus, previous studies using other study designs had no negative effects of the control condition on the training effect. Another difference to previous studies is the timing of the intervention during hospitalization when patients had severe symptoms and cognitive impairments typical of underweight patients with AN (AWMF S3-Leitlinie Diagnostik und Therapie der Essstörungen, 2018). Improvement in these symptoms typically occurs only with weight normalization (AWMF S3-Leitlinie Diagnostik und Therapie der Essstörungen, 2018). Thus, the lack of improvement may be due to the intervention starting too early, with an average baseline BMI of 18.12 kg/m^2^. Later training at a higher weight or more sessions may yield more consistent, clinically relevant results.

### 4.3 Comparison of the influence of the intervention among the diagnoses

This is the first study to examine CBM for body image in adolescent inpatients with DEP, comparing results to patients with AN. Both groups benefited, though the control condition had a more negative effect in patients with AN, who did not benefit more from the intervention, as expected. As in prior studies (Delinsky and Wilson, 2006), patients with DEP showed reduced depressive symptoms (PHQ), even while participating in the hospital’s DEP-specific inpatient treatment. Comparing study periods (Group A/B) demonstrated that the intervention caused a significant symptom reduction. The results support previous research and demonstrate how body image training can be integrated transdiagnostically, as suggested by Rawana (2013). While body image disturbance is central to AN diagnosis, the intervention significantly impacted body image in patients with AN despite their lower response to feedback, difficulty changing behavior (Foerde and Steinglass, 2017), need for control, and rigid thinking (AWMF S3-Leitlinie Diagnostik und Therapie der Essstörungen, 2018). These disorder-specific differences may explain the varying training effects between diagnostic groups highlighting the intervention’s potential to modify body perception even in challenging clinical populations.

In addition to disorder-specific characteristics that influence body image and, consequently, the outcomes of the 2-AFC task, contextual factors such as social, cultural, economic, and environmental determinants of health behavior may differ between diagnostic groups (e.g., Lorant et al., 2003; Swanson et al., 2011) and shape body image, as outlined in the Introduction. Such factors may help explain both interindividual and diagnosis-specific differences in responses to the 2-AFC task and should be systematically addressed in future research.

### 4.4 Limitations

This pilot study aimed to test the paradigm’s feasibility in adolescents in a clinical context. As a pilot, crossover-designed study, only small group sizes were included, and although we informally asked adolescents of a comparable age group about avatar preference, this does not constitute a systematic validation; thus, the avatar originally developed for adults in the UK was neither culturally adapted nor validated for adolescents. In the 2-AFC task, the avatar was used to measure shifts in perceptual boundaries, while questionnaires assessed disorder-specific symptoms such as body dissatisfaction. Previous studies have shown that variation in the categorical boundary is associated with body dissatisfaction (Cornelissen et al., 2022). Future studies should therefore incorporate direct perceptual body image measures to complement questionnaire-based assessments of body dissatisfaction. Many patients were excluded due to the long duration of the study with a shorter planned hospital stay, acute suicidality or an obese BMI in the DEP group. Participants with an obese BMI were excluded to avoid reinforcing a potentially negative self-perception of being “fat.” Besides IQ, no demographic covariates or emotional and motivational factors were assessed or controlled for in the structural model. Future studies should therefore consider including additional variables such as parental income, education, personal health experiences, and emotional or motivational alignment with the task. Furthermore, the confrontation with body ideals and corrective feedback may have induced stress in some participants, which could be reflected in the dropout of two patients with depression. The compatibility of participation during the clinical setting was sometimes difficult. Despite many appointments and repetitions, the tasks were completed reliably. The control condition’s non-neutral effect was another limitation. The analysis slightly deviated from the preregistered plan on AsPpredicted (# 68371) to align with CONSORT guidelines for crossover studies. Importantly, no changes were made to the hypotheses or outcome variables. Deviations were limited to analytic procedures, specifically variables differed to appropriately reflect the crossover structure and instead of the preregistered mediation analysis, a linear regression was conducted, as the temporal order required for mediation could not be assumed in the crossover context. A repeated-measures ANOVA was added so the second hypothesis could still be answered clearly. To ensure data usability despite deviations, the carryover effect was analyzed. While the study was transparently preregistered, it was subsequently registered with the German Clinical Trials Register (DRKS), a WHO-recommended trial registry for Germany, in response to additional journal requirements (Trial Registration Number: DRKS00037169).

## 5 Conclusions and future directions

This pilot study showed that a 2-AFC CBM paradigm can significantly change the individual boundary for perceiving a body as “fat” in adolescents with AN or DEP over 10 days. As the first systematic control study in adolescents, it demonstrated the intervention’s transdiagnostic applicability, highlighting body image disturbances in DEP. Confirmatory feedback had a non-neutral effect, especially in patients with AN whose individual boundary for judging a body as “fat” was aligned with normal weight. Larger follow-up studies with, e.g., parallel group designs are needed to explore the CBM training’s impact on psychopathology in detail. Based on the results of this study and the S3 guideline (AWMF S3-Leitlinie Diagnostik und Therapie der Essstörungen, 2018), patients with AN should be included after weight normalization and feedback should serve as orientation and reinforcement of a healthy weight range, particularly for suggestible patients. This study confirmed body image as a transdiagnostic factor, emphasizing the need for further research on body image disturbances in DEP and the effects of confirmatory feedback on adolescent’s body image in clinical and everyday settings.

## Data Availability

The raw data supporting the conclusions of this article will be made available by the authors, without undue reservation. Of note, some of the data was pre-printed following a different study protocol regarding analytic approaches (preprint link: doi: 10.31219/osf.io/t9j8u).

## Glossary

AN: (atypical) anorexia nervosa
DEP: depression
2-AFC: 2-alternative forced choice
CBM: cognitive bias modification
BMI: body mass index
FU: follow up
IQ: intelligence quotient
BSQ: body shape questionnaire
PHQ: patient health questionnaire
DSM: diagnostic and statistical manual of mental disorders
SCID: structured clinical interview
ANIS: anorexia nervosa self-assessment inventory
SPSS: statistical package für social sciences
Catbound: Categorial boundary
ANOVA: analysis of variance
vs.: versus
e.g.: for example
