# Supplementary material for: Effect of cognitive bias modification training on body image dissatisfaction in adolescents with anorexia nervosa or depression—a pilot feasibility randomized controlled crossover study
Source: Front Psychol. 2025 Sep 26;16:1655064. doi: 10.3389/fpsyg.2025.1655064 (PMC12510976; doi:10.3389/fpsyg.2025.1655064)
Supplement: Supplementary file 1 [file Table_1.DOCX]

**Table A1: Additional questionnaires**

| **Description** | PACS - Physical Appearance Comparison Scale Since body image disorder is influenced by internalizing and social comparison processes, we used the five-item physical appearance comparison scale (PACS) (Thompson, Heinberg & Tantleff-Dunn, 1991) to measure the degree to which participants compared their physical appearance with others in various social situations. Participants rated the frequency with which they made comparisons on a 5-point Likert scale ranging from 1 to 5 (1=never, 5=always), with higher scores correlating highly with body dissatisfaction and eating disturbance (Thompson, Heinberg & Tantleff-Dunn, 1991). The German version of the PACS has good test-retest reliability and validity of body-related social comparison habits in women (Mölbert et al., 2017).  OBCS - Objectified Body Consciousness Scale The perceived sense of shame of not conforming to the culturally accepted ideal of beauty was measured with the translated version of the Objectified Body Consciousness Scale (OBCS) (McKinley & Hyde, 1996) using the scales Control Beliefs, Body Surveillance and Body Shame. Participants rated statements on a 7-point scale from 1 (strongly disagree) to 7 (strongly agree), with a “not applicable” option. When participants marked more than 25% of the items of a given scale as not applicable, the subscale was counted as missing due to the OBSC scoring instructions (McKinley & Hyde, 1996). Compared to the original English-language version, five additional items are recorded in the German-language version (Knauss, Paxton & Alsaker, 2008). The change sensitivity of the OBCS has not yet been demonstrated.  BISS - Body Image State Scale To capture changes in body image perception and mood, the Body Image State Scale (BISS) (Cash et al., 2002) was administered. Participants rated six items on a 9-point Likert scale from 1 (=extremely unsatisfied with my appearance) to 9 (=extremely satisfied with my appearance) with lower scores correlating with more negative evaluation and higher dissatisfaction with weight, body size, shape, physical attractiveness, and appearance. The BISS shows internal consistency, stability, and validity (Cash et al., 2002). |
| --- | --- |
| **Baseline Characteristics of Patients With Depression and Anorexia Nervosa** | \| Variable \| Depression \| \|  \| Anorexia Nervosa \| \|  \| DEP vs. AN \| \| --- \| --- \| --- \| --- \| --- \| --- \| --- \| --- \| \|  \| *M* \| *SD* \|  \| *M* \| *SD* \|  \| *p* \| \| PACS \| 15.26 \| 5.51 \|  \| 17.58 \| 3.45 \|  \| ns \| \| BISS \| 3.7 \| 1.75 \|  \| 2.29 \| 1.23 \|  \| 0.017 \| \| OBCS Control Beliefs \| 3.87 \| 0.78 \|  \| 4.30 \| 0.66 \|  \| ns \| \| OBCS Body Shame \| 3.37 \| 1.42 \|  \| 4.40 \| 0.71 \|  \| 0.016 \| \| OBCS Body Surveillance \| 3.96 \| 1.87 \|  \| 5.36 \| 0.41 \|  \| 0.008 \|   Note: PACS=Physical Appearance Comparison Scale, BISS=Body Image States Scale; OBCS=Objectified Body Consciousness Scale, M=mean, SD=Standard deviation, DEP vs. AN p=two- tailed p value for unpaired t-test comparing means of the depression with means of the anorexia nervosa group. |
| **Carryover and treatment effect** | \|  \|  \|  \| \| --- \| --- \| --- \| \|  \| carryover effect \| treatment effect \| \| ANIS \| 0.405 \| 0.678 \| \| BSQ \| 0.556* \| 0.562 \| \| PHQ \| 0.534 \| 0.147 \| \| Catbound short \| 0.586* \| <0.001 \| \| Cabound long \| 0.647* \| 0.001 \| \| PACS \| 0.125 \| 0.556 \| \| BISS \| 0.068 \| 0.854 \| \| OBCS Control Beliefs \| 0.611 \| 0.495 \| \| OBCS Body Shame \| 0.454 \| 0.915 \| \| OBCS Body Surveillance \| 0.235* \| 0.777* \| \| Note: ANIS=Anorexia nervosa Inventory, BSQ=Body Shape Questionnaire, PHQ=Patient Health Questionnaire, PACS=Physical Appearance Comparison Scale, BISS=Body Image States Scale, OBCS=Objectified Body Consciousness Scale, *=Mann-Whitney U-test. \| \| \| |
| **Psychometric Task Performance in Depression and Anorexia Nervosa Groups** | \| Measure \| Depression \| \| \| \| \| \| \| \| \|  \| Anorexia nervosa \| \| \| \| \| \| \| \| \| \| --- \| --- \| --- \| --- \| --- \| --- \| --- \| --- \| --- \| --- \| --- \| --- \| --- \| --- \| --- \| --- \| --- \| --- \| --- \| --- \| \|  \| Group A \| \| \| \|  \| Group B \| \| \| \|  \| Group A \| \| \| \|  \| Group B \| \| \| \| \|  \| *M* \| \| *SD* \| \|  \| *M* \| \| *SD* \| \|  \| *M* \| \| *SD* \| \|  \| *M* \| \| *SD* \| \| \| PACS 1 \| 14.33 \| \| 5.92 \| \|  \| 16.29 \| \| 5.21 \| \|  \| 17 \| \| 3.46 \| \|  \| 18.75 \| \| 3.59 \| \| \| PACS 2 \| 14.5 \| \| 6.09 \| \|  \| 16.57 \| \| 5.57 \| \|  \| 16.75 \| \| 3.62 \| \|  \| 21 \| \| 2.94 \| \| \| PACS 3 \| 13.64 \| \| 5.96 \| \|  \| 14.1 \| \| 5.43 \| \|  \| 15.35 \| \| 4.06 \| \|  \| 21 \| \| 4.83 \| \| \| BISS 1 \| 3.91 \| \| 1.64 \| \|  \| 3.47 \| \| 1.95 \| \|  \| 2.4 \| \| 1.39 \| \|  \| 2.08 \| \| .96 \| \| \| BISS 2 \| 3.82 \| \| 1.57 \| \|  \| 3.19 \| \| 1.59 \| \|  \| 2.83 \| \| 1.62 \| \|  \| 1.54 \| \| .5 \| \| \| BISS 3 \| 4.34 \| \| 1.88 \| \|  \| 3.54 \| \| 2.05 \| \|  \| 3.08 \| \| 1.33 \| \|  \| 1.79 \| \| .95 \| \| \| OBCS Control Beliefs 1 \| 4.09 \| \| .54 \| \|  \| 3.62 \| \| .95 \| \|  \| 4.26 \| \| .79 \| \|  \| 4.41 \| \| .36 \| \| \| OBCS Control Beliefs 2 \| 4.19 \| \| .38 \| \|  \| 4.03 \| \| .55 \| \|  \| 4.08 \| \| .82 \| \|  \| 4.17 \| \| .27 \| \| \| OBCS Control Beliefs 3 \| 4.22 \| \| .53 \| \|  \| 3.91 \| \| .6 \| \|  \| 4.12 \| \| .86 \| \|  \| 4.19 \| \| .85 \| \| \| OBCS Body Shame 1 \| 3.16 \| \| 1.5 \| \|  \| 3.6 \| \| 1.38 \| \|  \| 4.28 \| \| .78 \| \|  \| 4.66 \| \| .54 \| \| \| OBCS Body Shame 2 \| 3.24 \| \| 1.54 \| \|  \| 3.65 \| \| 1.21 \| \|  \| 4.09 \| \| .93 \| \|  \| 4.91 \| \| 1.06 \| \| \| OBCS Body Shame 3 \| 2.95 \| \| 1.91 \| \|  \| 3.72 \| \| 1.01 \| \|  \| 4.05 \| \| .67 \| \|  \| 4.16 \| \| 1.04 \| \| \| OBCS Body Surveillance 1 \| 3.04 \| \| 2.01 \| \|  \| 5.01 \| \| 1.04 \| \|  \| 5.49 \| \| .46 \| \|  \| 5.09 \| \| .06 \| \| \| OBCS Body Surveillance 2 \| 3.04 \| \| 1.78 \| \|  \| 5.05 \| \| .54 \| \|  \| 5.28 \| \| .48 \| \|  \| 5.28 \| \| .77 \| \| \| OBCS Body Surveillance 3 \| 3.45 \| \| 1.73 \| \|  \| 4.95 \| \| .89 \| \|  \| 5.1 \| \| .5 \| \|  \| 5 \| \| .43 \| \| \|  \| \|  \| \|  \| \| \|  \| \|  \| \| \|  \| \|  \| \| \|  \| \|  \| \| \| Note: PACS=Physical Appearance Comparison Scale, BISS=Body Image States Scale, OBCS=Objectified Body Consciousness Scale, M=mean, SD=standard deviation. \| \| \| \| \| \| \| \| \| \| \| \| \| \| \| \| \| \| \| \| \| |
| **Regression Analyses Predicting Outcomes by Diagnosis** | \| Measure \| Depression \| \| \| \|  \| Anorexia nervosa \| \| \| \| \| --- \| --- \| --- \| --- \| --- \| --- \| --- \| --- \| --- \| --- \| \|  \| F(1,16) \| p \| R² \| Adj. R² \|  \| F(1,11) \| p \| R² \| Adj. R² \| \| BISS 1 \| 1.213 \| .288 \| .075 \| .013 \|  \| 9.305 \| .012 \| .482 \| .43 \| \| BISS 2 \| .999 \| .333 \| .062 \| 0 \|  \| .48 \| .504 \| .046 \| -0.05 \| \| PACS 1 \| 5.452 \| .034 \| .267 \| .218 \|  \| 2.388 \| .153 \| .193 \| .112 \| \| PACS 2 \| 5.709 \| .03 \| .276 \| .227 \|  \| 2.403 \| .152 \| .194 \| .113 \| \| OBCS Body Surveillance 1 \| .013 \| .91 \| .001 \| -0.066 \|  \| 1.704 \| .221 \| .146 \| .06 \| \| OBCS Body Surveillance 2 \| .395 \| .539 \| .026 \| -0.039 \|  \| .145 \| .712 \| .014 \| -0.084 \| \| OBCS Control Beliefs 1 \| .142 \| .711 \| .009 \| -0.057 \|  \| .395 \| .544 \| .038 \| -0.058 \| \| OBCS Control Beliefs 2 \| .648 \| .434 \| .041 \| -0.023 \|  \| .216 \| .652 \| .021 \| -0.077 \| \| OBCS Body Shame 1 \| 4.067 \| .062 \| .462 \| .161 \|  \| 5.433 \| .042 \| .352 \| .287 \| \| OBCS Body Shame 2 \| 1.34 \| .265 \| .286 \| .021 \|  \| .084 \| .778 \| .008 \| -0.091 \|   Note: PACS=Physical Appearance Comparison Scale, BISS=Body Image States Scale, OBCS=Objectified Body Consciousness Scale. |
| **Treatment Effects in Depression and Anorexia Nervosa Groups** | \| Measure \| Depression \| \| \| \|  \| Anorexia nervosa \| \| \| \| \| --- \| --- \| --- \| --- \| --- \| --- \| --- \| --- \| --- \| --- \| \|  \| Group A \| Group B \|  \|  \|  \| Group A \| Group B \|  \|  \| \|  \| *M* \| *M* \| *p* \| Cohen’s d \|  \| *M* \| *M* \| *p* \| Cohen’s d \| \| PACS \| .85 \| 2.47 \| .16 \| -0.73 \|  \| 1.40 \| 0 \| .37* \| .63 \| \| BISS \| -0.52 \| -0.35 \| .96* \| .02 \|  \| -0.24 \| -0.25 \| .99 \| 0 \| \| OBCS Control Beliefs \| -0.04 \| .12 \| .48* \| .37 \|  \| -0.05 \| -0.01 \| .94 \| -0.05 \| \| OBCS Body Shame \| .29 \| -0.07 \| .38 \| .43 \|  \| .04 \| .75 \| .08 \| -1.00 \| \| OBCS Body Surveillance \| -0.42 \| .10 \| .61* \| .26 \|  \| .18 \| .28 \| .80 \| .82 \|   Note: PACS=Physical Appearance Comparison Scale, BISS=Body Image States Scale, OBCS=Objectified Body Consciousness Scale, * = Mann-Whitney U-test (exact significance), Guidelines for interpretation of Cohen’s d indicate that 0.2 is a small effect, 0.5 is a medium effect and 0.8 is a large effect (67), indicating that the bold effect sizes are medium and large. |

**References:**

**Cash, T. F., Fleming, E. C., Alindogan, J., Steadman, L., & Whitehead, A. (2002).**Beyond body image as a trait: The development and validation of the body image states scale. *Eating Disorders, 10*(2), 103–113. https://doi.org/10.1080/10640260290081678

**Knauss, C., Paxton, S. J., & Alsaker, F. D. (2008).**Body dissatisfaction in adolescent boys and girls: Objectified body consciousness, internalization of the media body ideal and perceived pressure from media. *Sex Roles, 59*(9-10), 633–643. https://doi.org/10.1007/s11199-008-9474-7

**McKinley, N. M., & Hyde, J. S. (1996).**
The objectified body consciousness scale. *Psychology of Women Quarterly, 20*(2), 181–215.

**Mölbert, S. C., Hautzinger, M., Karnath, H. O., Zipfel, S., & Giel, K. E. (2017).**
Validierung der deutschsprachigen Version der Physical Appearance Comparison Scale (PACS): Psychometrische Eigenschaften und Zusammenhang mit Essverhalten, Körperbild und Selbstwert. *Psychotherapie, Psychosomatik, Medizinische Psychologie, 67*(2), 91–97. https://doi.org/10.1055/s-0042-123842

**Thompson, J. K., Heinberg, L., & Tantleff-Dunn, S. (1991).**
The Physical Appearance Comparison Scale. *Psychology Faculty Publications*, (2116). Retrieved from https://digitalcommons.usf.edu/psy_facpub/2116.
