# Supplementary material for: Effect of cognitive bias modification training on body image dissatisfaction in adolescents with anorexia nervosa or depression—a pilot feasibility randomized controlled crossover study
Source: Front Psychol. 2025 Sep 26;16:1655064. doi: 10.3389/fpsyg.2025.1655064 (PMC12510976; doi:10.3389/fpsyg.2025.1655064)
Supplement: Supplementary file 2 [file Table_2.DOCX]

| Table A2. Carryover and treatment effect | |  |
| --- | --- | --- |
|  |  |  |
|  | carryover effect | treatment effect |
| ANIS | .405 | .678 |
| BSQ | .556* | .562 |
| PHQ  Catbound short  Catbound long | .534  .586*  0.647* | .147  <.001  .001 |
|  |  |  |
| Note: ANIS=Anorexia nervosa Inventory, BSQ=Body Shape Questionnaire, PHQ=Patient Health Questionnaire, *=Mann-Whitney U-test | | |
