# Supplementary material for: Effect of cognitive bias modification training on body image dissatisfaction in adolescents with anorexia nervosa or depression—a pilot feasibility randomized controlled crossover study
Source: Front Psychol. 2025 Sep 26;16:1655064. doi: 10.3389/fpsyg.2025.1655064 (PMC12510976; doi:10.3389/fpsyg.2025.1655064)
Supplement: Supplementary file 3 [file Table_3.DOCX]

Table A3. Corresponding mean values of categorial boundary differences

|  |  |  |  | 1 | |  | 2 | |  | 3 | |  | 4 | |
| --- | --- | --- | --- | --- | --- | --- | --- | --- | --- | --- | --- | --- | --- | --- |
|  |  |  |  | *M* | *SD* |  | *M* | *SD* |  | *M* | *SD* |  | M | SD |
| AN A | Intervention | Pre |  | 0 | 0 |  | 0 | 1.07 |  | .13 | 1.24 |  | -0.13 | 1.55 |
|  |  | Post |  | .25 | 1.04 |  | .38 | 1.85 |  | .38 | 2.06 |  | .63 | 2 |
|  | Control | Pre |  | 0 | 0 |  | -0.42 | 0.5 |  | -0.77 | .68 |  | -1.13 | .83 |
|  |  | Post |  | -0.75 | .71 |  | -0.54 | .73 |  | -1.24 | .74 |  | -1.48 | 1.11 |
| B | Control | Pre |  | 0 | 0 |  | -2 | 1.4 |  | -3 | 1.15 |  | -3 | 2.58 |
|  |  | Post |  | -2.25 | 1.71 |  | -3 | 1.15 |  | -3.25 | 2.22 |  | -3.5 | 2.08 |
|  | Intervention | Pre |  | 0 | 0 |  | 0 | 1.41 |  | 0 | 1.83 |  | .25 | 1.5 |
|  |  | Post |  | .4 | .71 |  | -0.25 | 1.26 |  | .25 | 1.71 |  | .25 | 1.5 |
| DEP A | Intervention | Pre |  | 0 | 0 |  | .86 | 1.02 |  | .68 | 1.12 |  | .82 | 1.31 |
|  |  | Post |  | 1.33 | .87 |  | 1.84 | 1.72 |  | 1.23 | 1.48 |  | 1.84 | 1.84 |
|  | Control | Pre |  | 0 | 0 |  | -0.4 | .48 |  | -0.39 | .69 |  | -0.49 | 1.22 |
|  |  | Post |  | -0.39 | 1.11 |  | -0.39 | .86 |  | -0.01 | .71 |  | -0.59 | 1.32 |
| B | Control | Pre |  | 0 | 0 |  | .13 | 1.64 |  | .15 | 2.22 |  | .74 | 3.09 |
|  |  | Post |  | -0.13 | 1.64 |  | .38 | 2.72 |  | .68 | 3.62 |  | .74 | 3.65 |
|  | Intervention | Pre |  | 0 | 0 |  | .05 | .7 |  | .33 | 1.33 |  | .87 | 1.48 |
|  |  | Post |  | -0.01 | .76 |  | .43 | .99 |  | .9 | 1.56 |  | 1.17 | 2.09 |

Note: AN=Anorexia nervosa group, DEP=Depressive patient group, A=group A, B=group B, Pre=before training, Post=after training, M= mean values of the difference between post baseline of each training day and pre baseline of the training sequence plotted against zero, SD=Standard deviation.
